# Whole brain radiotherapy plus simultaneous in-field boost with image guided intensity-modulated radiotherapy for brain metastases of non-small cell lung cancer

**DOI:** 10.1186/1748-717X-9-117

**Published:** 2014-05-21

**Authors:** Lin Zhou, Jia Liu, Jianxin Xue, Yong Xu, Youling Gong, Lei Deng, Shichao Wang, Renming Zhong, Zhenyu Ding, You Lu

**Affiliations:** 1 Department of Thoracic Cancer, Cancer Center, West China Hospital of Sichuan University, Chengdu, Sichuan Province, China; 2 Department of Oncology, Chengdu First People’s Hospital, Chengdu, Sichuan Province, China; 3 Center of Radiation Physics, Cancer Center, West China Hospital of Sichuan University, Chengdu, Sichuan Province, China; 4 State Key Laboratory of Biotherapy, West China Hospital of Sichuan University, Chengdu, Chengdu, Sichuan Province, China

**Keywords:** Whole brain radiotherapy, Simultaneous in-field boost, Brain metastases, Non-small cell lung cancer

## Abstract

**Background:**

Whole brain radiotherapy (WBRT) plus sequential focal radiation boost is a commonly used therapeutic strategy for patients with brain metastases. However, recent reports on WBRT plus simultaneous in-field boost (SIB) also showed promising outcomes. The objective of present study is to retrospectively evaluate the efficacy and toxicities of WBRT plus SIB with image guided intensity-modulated radiotherapy (IG-IMRT) for inoperable brain metastases of NSCLC.

**Methods:**

Twenty-nine NSCLC patients with 87 inoperable brain metastases were included in this retrospective study. All patients received WBRT at a dose of 40 Gy/20 f, and SIB boost with IG-IMRT at a dose of 20 Gy/5 f concurrent with WBRT in the fourth week. Prior to each fraction of IG-IMRT boost, on-line positioning verification and correction were used to ensure that the set-up errors were within 2 mm by cone beam computed tomography in all patients.

**Results:**

The one-year intracranial control rate, local brain failure rate, and distant brain failure rate were 62.9%, 13.8%, and 19.2%, respectively. The two-year intracranial control rate, local brain failure rate, and distant brain failure rate were 42.5%, 30.9%, and 36.4%, respectively. Both median intracranial progression-free survival and median survival were 10 months. Six-month, one-year, and two-year survival rates were 65.5%, 41.4%, and 13.8%, corresponding to 62.1%, 41.4%, and 10.3% of intracranial progression-free survival rates. Patients with Score Index for Radiosurgery in Brain Metastases (SIR) >5, number of intracranial lesions <3, and history of EGFR-TKI treatment had better survival. Three lesions (3.45%) demonstrated radiation necrosis after radiotherapy. Grades 2 and 3 cognitive impairment with grade 2 radiation leukoencephalopathy were observed in 4 (13.8%) and 4 (13.8%) patients. No dosimetric parameters were found to be associated with these late toxicities. Patients received EGFR-TKI treatment had higher incidence of grades 2–3 cognitive impairment with grade 2 leukoencephalopathy.

**Conclusions:**

WBRT plus SIB with IG-IMRT is a tolerable and effective treatment for NSCLC patients with inoperable brain metastases. However, the results of present study need to be examined by the prospective investigations.

## Background

Non-small cell lung cancer (NSCLC) is one of the most common malignant tumors, and approximately 36%-44% of patients with NSCLC present with brain metastases during the course of disease [1]. During the past 50 years, whole brain radiotherapy (WBRT) has been the standard treatment for brain metastases, but its therapeutic effects are suboptimal with intracranial control rate (ICR) of 60% and median survival of 3–6 months [1,2]. Stereotactic radiosurgery (SRS) is beneficial in patients with limited number and volume of metastases and it has become increasingly available as an alternative focal treatment to surgery, but its therapeutic effects decrease with increasing number and volume of lesions [3]. Furthermore, SRS is not recommended in patients with lesions located in or close to critical anatomic structures because of unacceptable risk of severe long-term damage [3]. Hypofractionated stereotactic radiotherapy (hfSRT) combines the precise beam delivery of radiosurgical technique with the radiobiological advantages of fractionation, and has shown the results comparable to SRS [4].

Noncoplanar arcs, noncoplanar fixed fields and intensity modulation are the most frequently used stereotactic radiotherapy techniques. It has been reported that intensity-modulated radiotherapy (IMRT) technique results in improved dose conformity as compared to the other two techniques for the hemisphere and irregular tumor targets, and may increase the therapeutic ratio of treating large and/or irregularly shaped intracranial lesions [5]. Image guided intensity-modulated radiotherapy (IG-IMRT) is a new technique of radiotherapy. It improves the accuracy of treatment delivery by using cone beam computed tomography (CBCT) with x-ray volumetric images (XVI) to give the 3-dimensional anatomic information in the treatment position and to reduce setup uncertainty [6]. With these advantages, it is possible to administer hfSRT with IG-IMRT to brain metastases with non-invasive head fixation such as thermoplastic mask [6,7].

Several prospective trials proved the superiority of WBRT plus focal radiotherapy boost in patients with limited number and volume of brain metastases [8,9], and even in patients with a large number and volume of brain metastases, several studies indicated that focal hfSRT may be effective [10,11]. Radiotherapy schedules of most previous reports were WBRT plus sequential SRS/hfSRT boost. Recently, some reports showed that WBRT plus simultaneous in-field boost (SIB) with helical tomotherapy were effective and tolerable for brain metastases [12-15]. In our department, 40 Gy/20 f (5 f/week) used to be the standard schedule of WBRT, and some NSCLC patients with brain metastases received SIB with IG-IMRT in the fourth week of WBRT if they had no or only mild neurological symptoms during the first three weeks. Therefore, we conducted this retrospective study to evaluate the efficacy and toxicities of WBRT plus SIB with IG-IMRT for NSCLC patients with brain metastases.

## Methods

### Clinical information

From July 2006 to April 2009, 29 NSCLC patients with a total of 87 brain metastases were treated with WBRT plus SIB with IG-IMRT in our department. All of these patients had Karnofsky Performance Status (KPS) scores ≥50, and inoperable brain metastases determined by experienced neurosurgeons. Of these 29 patients, 11 received epidermal growth factor receptor tyrosine kinase inhibitor (EGFR-TKI) treatment (9 with gefinitib, 2 with erlotinib) concurrent with and maintained after radiotherapy. Detailed patient characteristics are summarized in Table 1. This retrospective study was approved and governed by the Review Board of West China Hospital of Sichuan University.

**Table 1:** Patient characteristics

**Characteristics**	**No. of patients(%)**
**Gender**	
Male	20 (68.97)
Female	9 (31.03)
**Age(years)**	
Median	58
Range	36-75
≥60	13 (44.83)
<60	16 (55.17)
**KPS scores**	
≥70	19 (65.52)
<70	10 (34.48)
**Pathology**	
Adenocarcinoma	18 (62.07)
Non-adenocarcinoma	11 (37.93)
**RPA class**	
2	19 (65.52)
3	10 (34.48)
**SIR scores**	
≤5	13 (44.83)
>5	16 (55.17)
**GPA scores**	
0-1	13 (44.83%)
1.5-2	11 (37.93%)
2.5-3	3 (10.34%)
3.5	2 (6.90%)
**History of EGFR-TKI treatment**	
Yes	11 (37.93)
No	18 (62.07)
**Number of lesions**	
Mean	3
<3	15 (51.72)
≥3	14 (48.28)
**Maximum lesion volume**	
<3 cc	12 (41.38)
≥3 cc	17 (58.62)
**Total volume of lesions**	
<7 cc	15 (51.72)
≥7 cc	14 (48.28)
**Total**	29

Abbreviations: KPS = Karnofsky performance status; RPA = recursive partitioning analysis; SIR = score index for radiosurgery in brain metastases; GPA = graded prognostic assessment; EGFR-TKI = epidermal growth factor receptor tyrosine kinase inhibitor.

### Treatment planning and delivery

**Figure 1:**
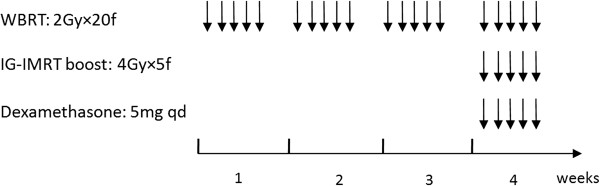
**Treatment schedule.** All patients received WBRT at a dose of 40 Gy/20 f/4 weeks, and SIB with IG-IMRT at a dose of 20 Gy/5 f concurrent with WBRT in the fourth week. Steroids (dexamethasone) were administered to all patients in the fourth week of radiotherapy. Abbreviations: WBRT = whole brain radiotherapy; IG-IMRT = image guided intensity-modulated radiotherapy; treatment delivery.

All patients received WBRT at a dose of 40 Gy/20 f (5 f/week), and SIB with IG-IMRT at a dose of 20 Gy/5 f concurrent with WBRT in the fourth week. Under this schedule of radiotherapy, intracranial lesions had received total boost dose of 30 Gy/5 f, and the biological effective dose (BED) value based on linear-quadratic (LQ) model (BED = nd [1 + d/(α/β)], α/β = 10 Gy) was close to the SRS boost of 15-18Gy/1f used to apply in our department. All patients were immobilized in non-invasive tight thermoplastic head masks (Med-Tec, U.S.A) and were treated by Synergy linear accelerator (Elekta, Sweden) with 6MV X-ray. Helical CT images of 3 mm slice thickness (Sensation 4, Siemens, Germany) were obtained by using the Precise Plan System (Version 2.11, Elekta, Sweden) and were fused with the previously generated MRI of 3 mm slice thickness (sonata maestro class, Siemens, Germany) by the image fusion system software. Clinical target volume (CTV) was defined as the whole brain. Planning target volume (PTV) was defined by adding a 3D isotropic margin of 5 mm to the CTV according to set-up inaccuracy. Gross tumor volume of lesion (GTV-L) was defined as the contrast enhancing tumor on MRI T1 scans. CTV of lesion (CTV-L) was defined as identical with GTV-L. PTV of lesion (PTV-L) was defined by adding a 3D isotropic margin of 2 mm to the GTV-L according to set-up inaccuracy. Inverse coplanar IMRT planning was used to ensure that 95% of PTV was covered by 100% isodose envelope of 40 Gy, and 100% of PTV-L was covered by 100% isodose envelope of 60 Gy. All patients underwent on-line positioning verification and correction to ensure that the set-up errors were within 2 mm by XVI 3D VolumeView imaging CBCT (Elekta, Sweden) prior to each IG-IMRT boost. Planning CT and VolumeView images were registered using automatic image registration (bone match) in the XVI software. Steroids were administered to all patients in the fourth week of radiotherapy and if patients were symptomatic in the first three weeks (see Figure 1 for the treatment schedule). All patients had completed the treatment successfully.

### Follow-up and statistics

All patients were subjected to weekly neurologic examination during the radiotherapy, and underwent clinical follow-up examinations including contrast-enhanced MRI 1 month after the end of radiotherapy and every 3 months thereafter. Response evaluation was based on the World Health Organization (WHO) criteria, and assessed by measurement of enhanced lesions in T1-weighted contrast-enhanced MRI image. ICR was defined as rates of complete response (CR) plus partial response (PR) and stable disease (SD) of intracranial lesions. Objective response rate (ORR) was defined as rates of CR plus PR of intracranial lesions. Local brain failure rate (LBFR) was defined as rate of progression of previously treated brain metastases. Regional brain failure rate (RBFR) was defined as rate of new brain metastases. Overall survival time (OS) was calculated from the day of starting radiotherapy to the last day of follow-up or death. The intracranial progression-free survival (IC-PFS) was calculated from the day of starting radiotherapy to the day of intracranial lesions progression, death or the last day of follow-up. All side effects were evaluated according to the National Cancer Institute Common Terminology Criteria for Adverse Events (CTCAE) ver. 3.0 grading system. Side effects occurring beyond 90 days from the end of radiotherapy were considered late toxicities.

The Kaplan-Meier method was used to estimate survival curve, ICR, LBFR, and RBFR. The Log-rank test was used for univariate analysis of prognostic factors, and variables found significant in univariate analysis were further subjected to multivariate Cox-regression. The crosstabs was used for univariate analysis of the risk factors of late toxicities, and variables found significant in univariate analysis were further subjected to multivariate binary logistic regression. The independent-samples t-test was used for the dosimetry analysis. *p* < 0.05 was regarded as statistically significant. All calculations were performed by SPSS 17.0.

## Results

### Local tumor control and survival

**Figure 2:**
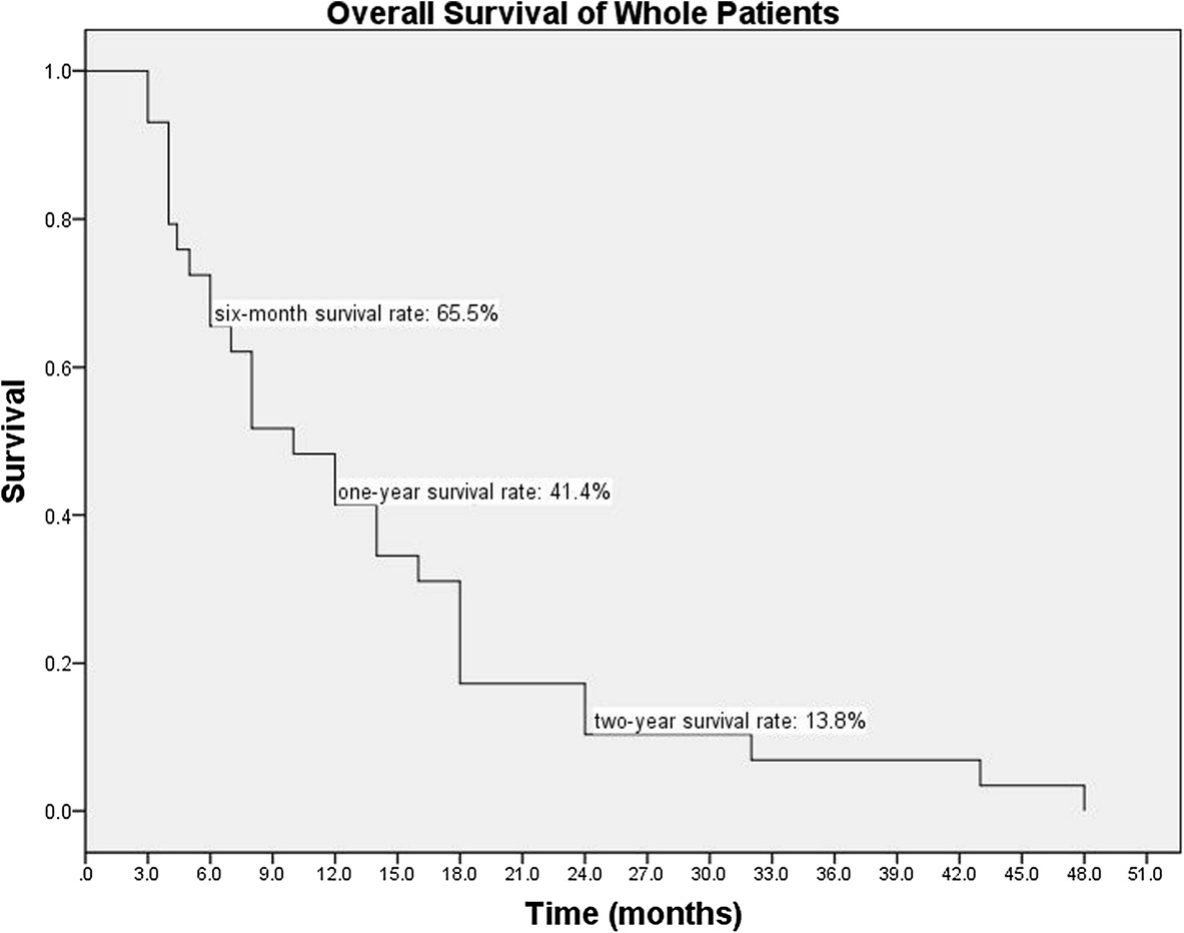
**Overall survival of the whole patients.** The Kaplan-Meier overall survival curve of whole group showed the six-month, one-year and two-year survival rates were 65.5%, 41.4% and 13.8%, respectively.

**Figure 3:**
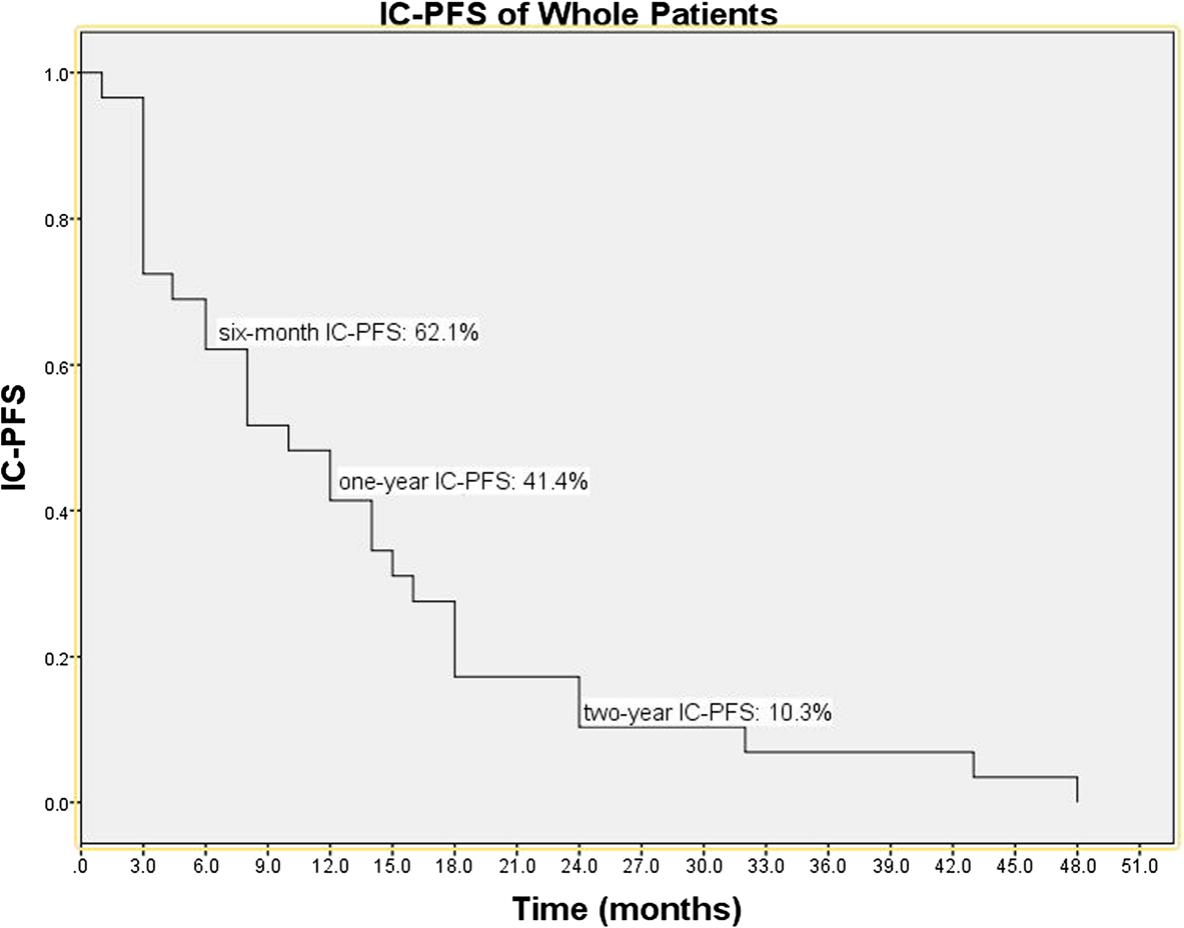
**IC-PFS of the whole patients.** The Kaplan-Meier IC-PFS curve of whole group showed the six-month, one-year and two-year IC-PFS rates were 62.1%, 41.4% and 10.3%, respectively. Abbreviations: IC-PFS = intracranial progression-free survival.

The median follow-up time was 10 months (range: 3–48 months), 27.6% (8/29) of patients died within 6 months after radiotherapy, and 72.4% (21/29) of patients were followed-up beyond 6 months. Up to the last follow-up visit, 13 patients had intracranial failure, with 7 new intracranial metastases and 6 locoregional progression. The ORR was 69%. The one-year ICR, LBFR, and RBFR were 62.9%, 13.8%, and 19.2%, respectively. The two-year ICR, LBFR, and RBFR were 42.5%, 30.9%, and 36.4%, respectively. Twenty-one patients died due to extracranial failure and 8 died due to intracranial failure. The OS of whole patients ranged from 3 to 48 months, and the median survival (MS) was 10 months (95% CI: 5.6-14.4 months). The six-month, one-year, and two-year survival rates were 65.5%, 41.4%, and 13.8%, respectively (Figure 2). The IC-PFS of whole patients ranged from 1 to 43 months, and the median IC-PFS was 10 months (95% CI: 4.7-15.3 months). The six-month, one-year, and two-year IC-PFS rates were 62.1%, 41.4%, and 10.3%, respectively (Figure 3).In univariate analysis, female, adenocarcinoma, Score Index for Radiosurgery in Brain Metastases (SIR) score >5, number of intracranial lesions <3, total volume of intracranial lesions <7 cc, and history of EGFR-TKI treatment were significant predictors for better OS and IC-PFS(see Table 2). In multivariate analysis, SIR score >5, number of intracranial lesions <3, and history of EGFR-TKI treatment remained significant as favorable prognostic factors (see Table 2).

**Table 2:** Univariate and multivariate analysis of IC-PFS, median survival and incidence of grade 2 leukoencephalopathy

	**IC-PFS (m) Median**	**Univariate *p*-value**	**Multivariate *p*-value**	**MS(m)**	**Univariate *p*-value**	**Multivariate *p*-value**	**Leukoencephalopathy No. of patients (%)**	**Univariate *p*-value**	**Multivariate *p*-value**
**Gender**									
Male	6	0.020	0.869	7	0.019	0.983	3/20 (15.5)	0.024	0.562
Female	18			18			5/9 (55.6%)		
**Age** (years)									
≥60	8	0.628		8	0.719		5/18 (27.8)	0.794	
<60	12			12			3/11 (27.3)		
**KPS scores**									
≥70	12	0.251		12	0.223		5/19 (26.3)	0.833	
<70	3			4.4			3/10(30)		
**Pathology**									
Adenocarcinoma	14	0.015	0.519	14	0.011	0.553	6/13 (46.2)	0.044	0.084
Non-adenocarcinoma	3			4.4			2/16 (12.5)		
**RPA class**									
2	12	0.266		12	0.185		5/19 (26.3)	0.833	
3	3			4.4			3/10 (30.0%)		
**GPA scores**									
0-1	6	0.331		7	0.188		4/13 (30.8)	0.730	
1.5-3.5	14			14			4/16 (25%)		
**SIR scores**									
≤5	3	0.001	0.044	4.4	<0.001	0.021	5/13 (38.5)	0.238	
>5	14			14			3/16 (18.75)		
**History of EGFR-TKI treatment**									
Yes	16	0.026	0.035	18	0.022	0.040	7/11 (63.6)	0.001	0.010
No	6			7			1/18 (5.6)		
**Number of lesions**									
<3	14	0.005	0.036	14	0.007	0.048	6/15 (40.0)	0.122	
≥3	3			6			2/14 (14.3)		
**Maximum lesion volume**									
<3 cc	14	0.209		14	0.081		4/12 (33.3)	0.561	
≥3 cc	4.4			6			4/17 (23.5)		
**Total volume of lesions**									
<7 cc	14	0.032	0.232	14	0.037	0.256	5/15 (33.3)	0.474	
≥7 cc	3			6			3/14 (21.4)		
**Total**	10			10			8/29 (27.6)		

Abbreviations: IC-PFS = intracranial progression-free survival; MS = median survival; m = month; RPA = recursive partitioning analysis; GPA = graded prognostic assessment; SIR = score index for radiosurgery in brain metastases; EGFR-TKI = epidermal growth factor receptor- tyrosine kinase inhibitor.

### Toxicities

The radiation-induced acute toxicities were generally mild (grades 1–2). The most frequent toxicities were grade 2 alopecia (reported in 29 patients, 100%), grade 1 radiation dermatitis (reported in 29 patients, 100%), grades 1–2 headache (reported in 8 patients, 27.6%), and grades 1–2 nausea (reported in 5 patients, 17.2%). There were no grade 3 acute toxicities.

The radiation-induced late toxicities were reported in 25 patients, including grades 1 and 2 leukoencephalopathy in 17 (58.6%) and 8 (27.6%) patients, respectively; grades 1, 2, and 3 cognitive disturbance in 6 (20.7%), 4 (13.8%), and 4 (13.8%) patients, respectively. All patients with grade 2 or 3 cognitive impairment also had grade 2 leukoencephalopathy. Of 89 lesions, 3 (3.45%) lesions from 2 (6.90%) different patients demonstrated radiation necrosis after radiotherapy. In univariate analysis, female, age ≥ 60 year-old, and history of EGFR-TKI treatment were risk factors for grade 2 leukoencephalopathy, and only history of EGFR-TKI treatment remained statistically significant in multivariate analysis (see Table 2). The time from radiotherapy to the occurrence of grade 2 or 3 cognitive impairment was 2–24 months with the median time of 9 months. The time from radiotherapy to the occurrence of grade 1 or 2 radiation leukoencephalopathy was 2–9 months with the median time of 6 months.

### Dosimetry

The median GTV of individual lesions were 6.62 cc (range: 2.41 cc-70.74 cc), and the median whole brain volume were1420.9 cc (range: 1215.2 cc-1587.5 cc). At the time of IG-IMRT boost, some region of normal brain tissue would have received more than 3-5 Gy/f. VXG was defined as volume of normal brain tissue receiving at least XGy per fraction during the SIB, and the V3G, V4G, and V5G in present study ranged from 47.90 cc to 474.30 cc, 15.40 cc to 241.26 cc, and 8.36 cc to 128.30 cc, respectively. VX was defined as the percentage volume of normal brain receiving at least XGy per fraction during the SIB. The V3, V4, and V5 in present study ranged from 1.11% to 10.87%, 0.37% to 5.46%, and 0.20% to 2.98%, respectively. In the dosimetry analysis, there was no correlation between the incidence of grade 2 leukoencephalopathy and V3G-V5G or V3-V5 (see Table 3). However, number of intracranial lesions >3, maximum intracranial lesion volume ≥3 cc, and total volume of intracranial lesions ≥7 cc were statistically significant associated with larger V3G-V5G and V3-V5 (see Table 3).

**Table 3:** Univariateanalysis of V3G-V5G and V3-V5

	**V3G (cc)**		**V4G (cc)**		**V5G (cc)**		**V3 (%)**		**V4 (%)**		**V5 (%)**	
	**Mean ± SD**	***p***	**Mean ± SD**	***p***	**Mean ± SD**	***p***	**Mean ± SD**	***p***	**Mean ± SD**	***p***	**Mean ± SD**	***p***
**leukoencephalopathy**												
Grade 0-1	204.94 ± 150.85	0.288	77.94 ± 67.47	0.316	34.12 ± 30.99	0.574	4.66 ± 3.40	0.102	1.77 ± 1.52	0.334	0.78 ± 0.71	0.609
Grade 2	128.60 ± 80.62		51.08 ± 49.53		27.14 ± 24.85		3.00 ± 1.81		1.18 ± 1.12		0.63 ± 0.57	
**Number of lesions**												
<3	126.05 ± 115.60	0.017	39.06 ± 44.83	0.004	18.65 ± 17.00	0.010	2.94 ± 2.55	0.020	0.91 ± 0.98	0.004	0.44 ± 0.39	0.013
≥3	245.84 ± 136.82		104.24 ± 64.15		46.70 ± 32.90		5.57 ± 3.14		2.36 ± 1.47		1.05 ± 0.76	
**Maximum lesion volume**												
<3 cc	94.13 ± 67.73	0.004	35.77 ± 27.52	0.006	15.35 ± 7.55	0.003	2.20 ± 1.42	0.001	0.83 ± 0.61	0.006	0.36 ± 0.17	0.004
≥3 cc	238.73 ± 143.95		91.77 ± 70.05		42.49 ± 32.74		5.43 ± 3.24		2.08 ± 1.58		0.97 ± 0.75	
**Total volume of lesions**												
<7 cc	108.74 ± 76.57	0.002	35.82 ± 31.48	0.002	15.43 ± 11.06	0.001	2.55 ± 1.75	0.003	0.83 ± 0.70	0.002	0.36 ± 0.25	0.002
≥7 cc	264.39 ± 145.93		107.71 ± 68.56		50.15 ± 30.07		5.98 ± 3.30		2.44 ± 1.56		1.14 ± 0.74	
**Total**	183.87 ± 138.15		70.53 ± 63.36		32.19 ± 29.16		4.21 ± 3.10		1.61 ± 1.43		0.74 ± 0.67	

Abbreviations: VXG =. The volume of normal brain tissue receiving at least XGy per fraction during the SIB; VX =. The percentage volume of normal brain receiving at least XGy per fraction during the SIB; cc = cubic centimeter.

## Discussion

WBRT plus sequential focal SRS/hfSRT boost is one of the most widely used therapeutic strategies for patients with limited brain metastases. However, evidence on efficacy and toxicities of WBRT plus simultaneous hfSRT are emerging recently. The phase I trial of WBRT plus SIB with helical tomotherapy showed that the delivery of 60 Gy/10 f synchronously with WBRT of 30 Gy was tolerable in patients with 1–3 brain metastases, and the Phase II trial is ongoing to examine its efficacy [12,13]. Just like helical tomotherapy, the Synergy IGRT system used in our study is one of integrated image-guided intensity-modulated-capable radiotherapy platforms. However, different from the more advanced arc-based IMRT such as helical tomotherapy, the radiation technology used in our study was a step-and-shoot IMRT which was more common and economically feasible in developing countries. Furthermore, only NSCLC patients were eligible in our study, and they were more homogeneous with respect to previous reports of WBRT plus SIB with helical tomotherapy, which enrolled patients without prescribing a limit to pathological type of primary cancer [12-14].

The reported one-year ICR and median survivals of patients with limited brain metastases received WBRT plus sequential focal hfSRT boost were 66%-86% and 7.5-13 months [16-20]. Unlike reports above, approximately half of patients in our study had multiple (≥3) or large (≥3 cc) intracranial lesions, which were always excluded in other studies. However, the median survival and one-year ICR of whole patients in our study were 10 months and 62.9%, which were close to previous data in above studies. Several grading systems are available for brain metastases, such as Graded Prognostic Assessment (GPA), Recursive Partitioning Analysis (RPA) and SIR. Higher level of RPA and lower scores of GPA and SIR are associated with worse survival of patients with brain metastases [21-24]. Similarly, the survival was better in RPA class II, GPA scores 1.5-3.5, and SIR >5 patients in our study. However, possibly because of the limited number of enrolled patients, only differences in survivals between patients with SIR ≤5 and >5 were statistically significant. The number of lesions is another important factor affecting radiotherapy efficacy, and patients with limited number of brain metastases seem to have better outcomes. It has been reported that median survivals were 6.5-16 months following WBRT plus focal radiotherapy boost in patients with single intracranial lesion, corresponding to 5.8-13months in patients with multiple intracranial lesions [8,9,16,17]. Similarly, patients with number of lesions <3 showed better outcomes in our study, and the median survival was comparable to the data reported by other investigators [11,17,20].

Several studies showed that the radiosensitivity of brain metastases is associated with the mutation status of epidermal growth factor receptor (EGFR) [25-27]. Compared to patients with the wild-type, those with activating EGFR mutations had higher response rates and better survival following WBRT (54% *VS.* 24%, *P* = 0.045; 17.3 months *VS*. 6.6 months, *P* = 0.121) [26]. EGFR-TKI, either alone or combined with WBRT, is effective in brain metastases of NSCLC especially for EGFR-mutated patients [26,28,29]. From the phase I and II trials of WBRT with concurrent and maintenance erlotinib in NSCLC with brain metastases, erlotinib in combination with WBRT was well tolerated and had a favorable efficacy. Moreover, EGFR-mutated patients had a better survival compared to those with wild-type (median survival: 19.1 months *VS.* 9.3 months) [29,30]. In present study, history of EGFR-TKI treatment was also a favorable prognostic factor for survival, and the median survival of 11 patients received concurrent and maintenance EGFR-TKI treatment was as long as 18 months. EGFR mutation status is not available in our study. However, most patients who received EGFR-TKI in this study were female (72.7%) and had adenocarcinoma (81.8%). Considering the evidence that Asian, female, non-smoking, and adenocarcinoma patients were more likely to be EGFR mutated, and rates of EGFR mutation in brain metastases of NSCLC were 44%-63% in East Asian population [31,32]. We speculated that EGFR mutations might contribute to the better survival in the EGFR-TKI treated patients in present study.

Considering the concurrent WBRT, intracranial lesions received total boost doses of 30 Gy/5 f in our study. By using the α/β ratio of 12 Gy and LQC model (BED = nd [1 + d/(α/β)-d^2^/(α/γ)]), which was indicated to be suitable for calculating BED value of SRS or hfSRT for brain metastases [33], the BED value of total boost were 43.33 Gy, which was comparable to other reports of brain metastases treated by hfSRT [12,13,16-20,34]. Late radiation toxicity of 13.8% grade 3 cognitive impairment in our group was comparable to previous data of 6%-11% grade 3 late toxicities from other studies [16,17]. The incidence of radiation necrosis was one of the major concerns in late toxicities from WBRT plus focal radiotherapy boost, but it was quite infrequent in our study, and was similar to other reports of WBRT plus focal hfSRT boost [10,16-18]. Leukoencephalopathy and cognitive impairment were primary late toxicities in present study, and only history of EGFR-TKI treatment was a risk factor for grade 2 leukoencephalopathy in multivariate analysis, which had not been reported by other studies. Patients who received EGFR-TKI treatment had the longer survival, which might partly explain the higher incidence of late toxicities in these patients. It has been reported that the EGFR-TKI plus concomitant WBRT may have synergy effect in brain metastases from NSCLC, the ORR, ICR, and IC-PFS were significantly higher in gefitinib plus WBRT compared with gefitinib alone (ORR: 64.4% *VS.* 26.7%, *P* < 0.001; DCR: 71.1% *VS.* 42.2%, *P* = 0.006; IC-PFS: 10.6 months *VS.* 6.57 months, *P* < 0.001) [35]. It is a reasonable assumption that the EGFR-TKI plus concomitant brain radiotherapy might also have synergy neurotoxicity, and it might be another reason for the higher incidence of late toxicities in patients with EGFR-TKI treatment. However, considering the limited number of patients in our study, it may need more preclinical and clinical data to support this assumption.

This retrospective study had many limitations such as heterogeneity and limited number of enrolled patients, lack of phase I data, lack of health-related quality of life (HRQoL) data, and lack of neurocognative testing data. We look forward to that the ongoing phase II trial of SIB with helical tomotherapy for 1–3 brain metastases will give us more information [13].

## Conclusions

WBRT plus SIB with IG-IMRT is a tolerable and effective treatment for NSCLC patients with inoperable brain metastases, especially for those with SIR score >5, number of intracranial lesions <3, and history of EGFR-TKI treatment. However, the results of present study need to be examined by the prospective investigations.

## Abbreviations

BED: Biological effective dose; CBCT: Cone beam computed tomography; CR: Complete response; CTCAE: National cancer institute common terminology criteria for adverse events; CTV: Clinical target volume; CTV-L: Clinical target volume of lesion; EGFR: Epidermal growth factor receptor; EGFR-TKI: Epidermal growth factor receptor tyrosine kinase inhibitor; GPA: Graded prognostic assessment; GTV-L: Gross tumor volume of lesion; hfSRT: Hypofractionated stereotactic radiotherapy; HRQoL: Health-related quality of life; ICR: Intracranial control rate; IC-PFS: Intracranial progression-free survival; IMRT: intensity-modulated radiotherapy; IG-IMRT: Image guided intensity-modulated radiotherapy; KPS: Karnofsky performance status; LBFR: Local brain failure rate; LQ: Linear-quadratic; MS: Median survival; NSCLC: Non-small cell lung cancer; ORR: Objective response rate; OS: Overall survival; PR: Partial response; RPA: Recursive partitioning analysis; PTV: Planning target volume; PTV-L: Planning target volume of lesion; RBFR: Regional brain failure rate; SD: Stable disease; SIB: Simultaneous in-fieldboost; SIR: Score index for radiosurgery in brain metastases; SRS: Stereotactic radiosurgery; V3G: The volume of normal brain tissue received more than 3 Gy per fraction; V4G: The volume of normal brain tissue received more than 4 Gy per fraction; V5G: The volume of normal brain tissue received more than 5 Gy per fraction; V3: The percentage of normal brain volume exceeding 3 Gy per fraction; V4: The percentage of normal brain volume exceeding 4 Gy per fraction; V5: The percentage of normal brain volume exceeding 5 Gy per fraction; WBRT: Whole brain radiotherapy; WHO: World Health Organization; XVI: X-ray volumetric images.

## Competing interests

There is no actual or potential conflict of interest in this study.

## Authors’ contributions

LZ participated in its design and drafted the manuscript. JL helped to review medical records and draft the manuscript. JX helped to analyze the data of local tumor control and survival. YX helped to review medical records. YG and LD helped to draft the manuscript and participated in coordination. SW helped to analyze the data of dosimetry. RZ helped to analyze the data of dosimetry. ZD helped to review medical records. YL designed the study and participated in coordination. All authors read and approved the final manuscript.
